# Medical Literature Writing With ChatGPT: A Rare Case of Choriocarcinoma Syndrome With Hemorrhagic Brain Metastases Due to Burned Out Metastatic Mixed Testicular Cancer

**DOI:** 10.7759/cureus.36655

**Published:** 2023-03-24

**Authors:** David P Le, Samuel C Hall

**Affiliations:** 1 Internal Medicine, University of South Alabama, Mobile, USA

**Keywords:** pulmonary hemorrhage, embryonal cell carcinoma, choriocarcinoma, testicular cancer, germ cell and embryonal neoplasms, chatgpt, metastatic testicular cancer, burned-out testicular tumor, intracerebral hemorrhage, choriocarcinoma syndrome

## Abstract

Testicular tumors are one of the most commonly observed malignancies among men. An aggressive and rare subtype of the disease, testicular choriocarcinoma, has a worse prognosis due to the tendency of early hematogenous spread to multiple organs and advanced symptoms at presentation time. Characteristic features of choriocarcinoma include elevated beta human chorionic gonadotropin (β-hCG) in a young male with testicular mass. However, when the primary testicular tumor overutilizes its blood supply and spontaneously regresses, it is presumed to have been "burnt out" with remnants evident by metastatic retroperitoneal lymphadenopathy, scarred tissue, and calcifications. Treatment of advanced testicular cancer may be complicated by a rare entity known as choriocarcinoma syndrome, distinguished by rapid and fatal hemorrhaging of metastatic tumor sites. Prior described cases of choriocarcinoma syndrome involve pulmonary and gastrointestinal hemorrhages. We present an uncommon case of a 34-year-old male with a "burnt out" metastatic mixed testicular cancer who presented with choriocarcinoma syndrome (CS) treated with chemotherapy but developed deadly hemorrhaging of brain metastases. In addition, with the assistance of ChatGPT, we report our experience with this OpenAI tool and its potential uses in medical literature writing.

## Introduction

Testicular choriocarcinoma is a highly aggressive, rare subtype of non-seminomatous germ cell tumor. Pure choriocarcinoma comprises less than 1% of all testicular tumors, while mixed germ cell tumors are found in around 8% of the cases [1]. Early stages of choriocarcinoma involve hematogenous metastases to any tissue, including the lungs, brain, bone, skin, and liver [1]. The hallmark of the presentation of choriocarcinoma is typically in a young male with painless testicular mass with elevated beta human chorionic gonadotropin (β-hCG) levels [1]. However, a testicular exam and ultrasound may be unremarkable with advanced presentations, with only "burnt-out" evidence of the remnants of a prior testicular source [2]. Clinicians need to recognize that patients with advanced testicular cancer can have extensive multi-organ involvement, and the first presenting symptoms may be heralded as a medical emergency. Choriocarcinoma syndrome (CS) is a life-threatening complication of choriocarcinoma that may present with spontaneous hemorrhages from the metastatic sites. Vague symptoms such as headaches, vomiting, and hemoptysis should be signs and symptoms of systemic involvement of choriocarcinoma that prompt a clinician to start early treatment to change the course of a patient's outcome.

## Case presentation

**Figure 1:**
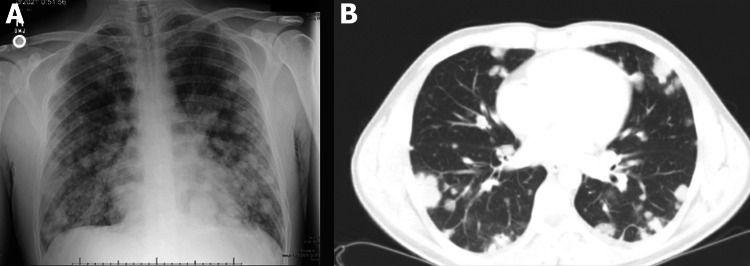
(A) Chest X-ray with diffuse pulmonary nodules and masses. (B) CT scan of diffuse pulmonary metastases. CT, computerized tomography

**Figure 2:**
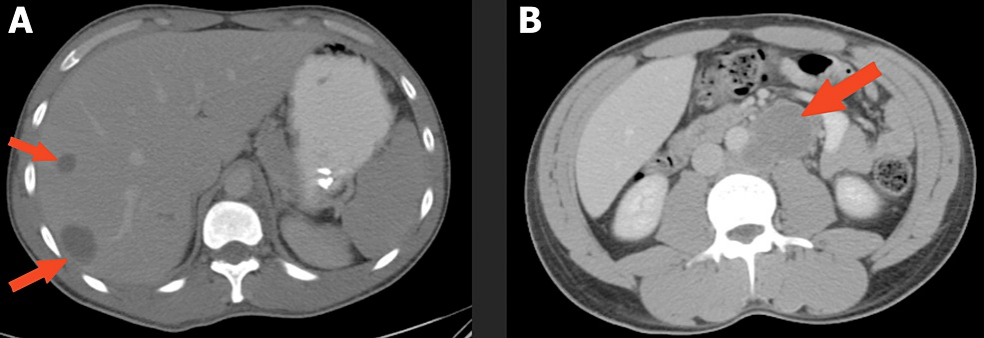
(A) CT abdomen with liver metastases. (B) CT with bulky retroperitoneal lymph nodes. CT, computerized tomography

A 34-year-old male initially presented for an episode of hemoptysis and dark stools. He also complained of persistent back, inguinal, and flank pain for three weeks. The patient denied fever, dyspnea, weakness, weight loss, hematochezia, hematuria, testicular mass, or pain. The physical exam was unrevealing, and the patient was vigilant, oriented, and with normal testicular palpation. Chest x-ray and computerized tomography (CT) of the chest showed numerous bilateral pulmonary nodules (Figure 1). CT abdomen and pelvis showed multiple metastatic lesions scattered throughout the liver and multiple enlarged retroperitoneal lymph nodes (Figure 2).

**Figure 3:**
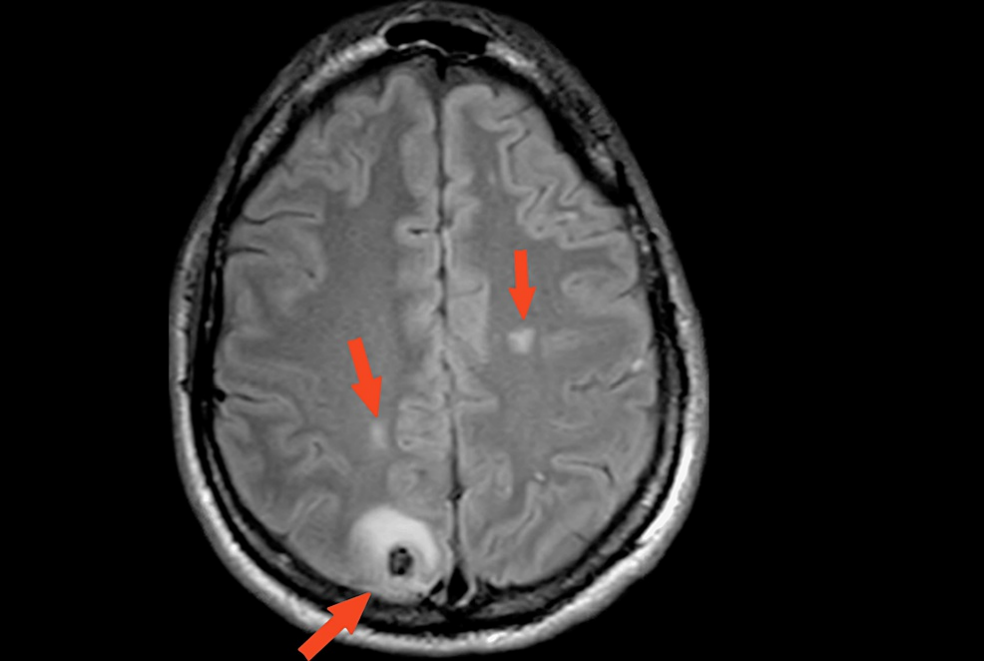
MRI Brain T2 FLAIR, hyperintensive lesions, largest located right posterior parietal lobe with perifocal edema consistent with blood product. MRI, Magnetic resonance imaging

Endobronchial ultrasound with biopsy of pulmonary nodules showed pathology consistent with germ cell neoplasm with features of both choriocarcinoma and embryonal carcinoma. MRI brain showed multiple metastases with a lesion demonstrating perifocal edema and blood product deposition (Figure 3).

**Figure 4:**
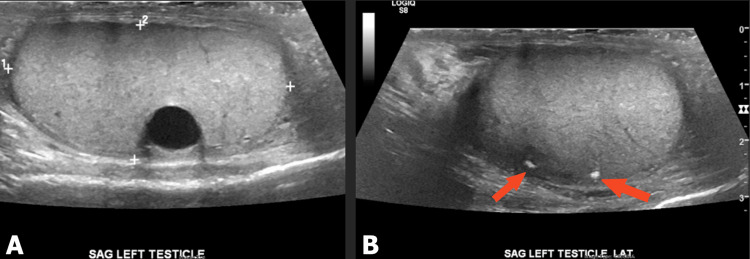
(A) Circumscribed structure, an echogenic scar with acoustic shadowing. (B) Calcifications in left testicle suggestive of a “burnt out” primary testicular tumor.

**Figure 5:**
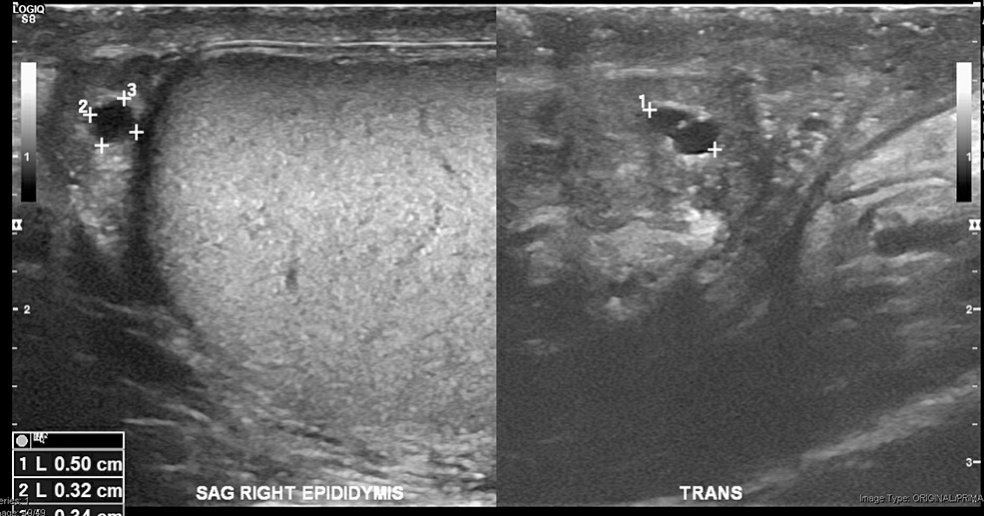
Right testicle with circumscribed, anechoic structure in the right epididymis demonstrating increased transmission.

**Figure 6:**
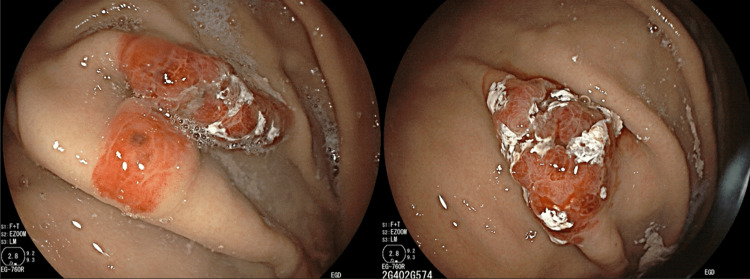
Multiple raised, irregular lesions throughout gastric body.

Testicular ultrasound showed no tumor source with apparent mass but a left testicle with scattered testicular calcifications and an echogenic scar seen as a circumscribed anechoic structure (Figure 4). A well-circumscribed, anechoic structure was also located in the right epididymis (Figure 5). Esophagogastroduodenoscopy revealed several erythematous, non-active bleeding lesions throughout the gastric body (Figure 6).

**Figure 7:**
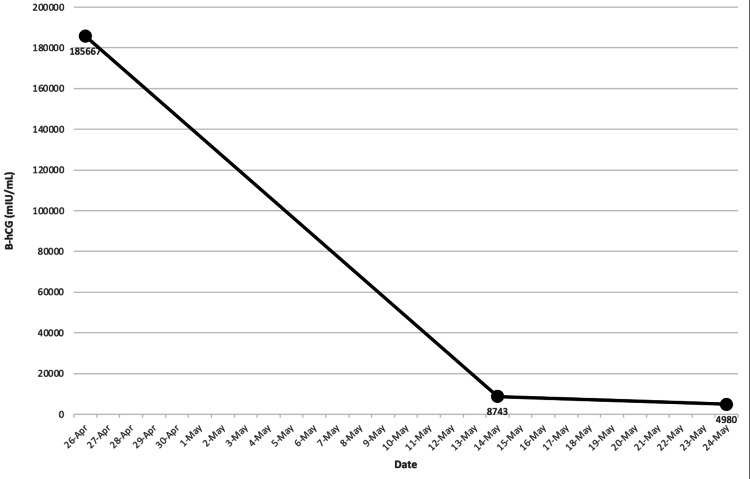
After two BEP cycles, serum B-hCG remained elevated at 4980 mIU/mL. BEP, bleomycin, etoposide, cisplatin. B-hCG, beta-human chorionic gonadotropin

**Figure 8:**
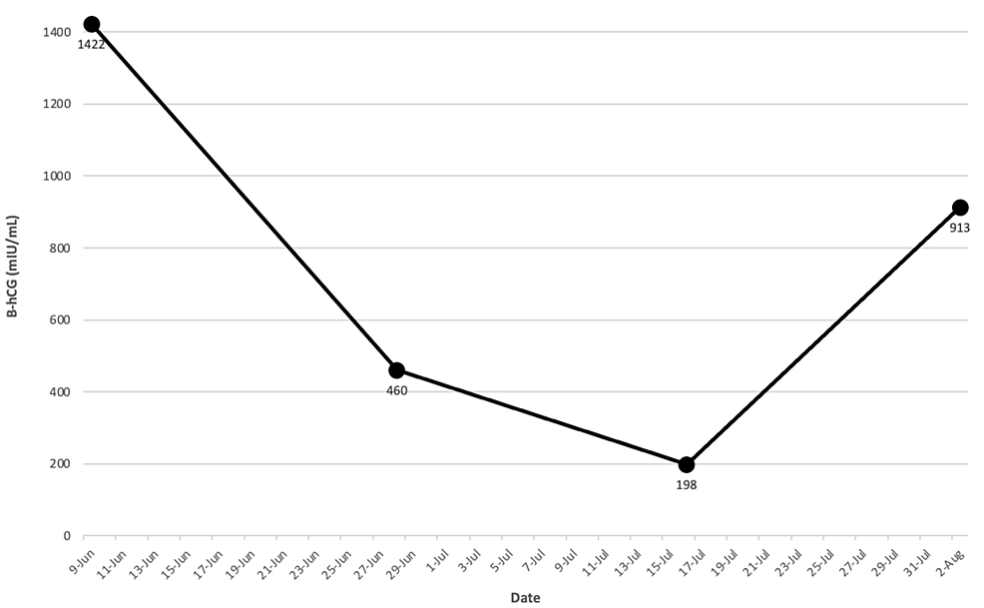
After four VIP cycles, serum B-hCG decreased to 198 mIU/mL, however two weeks after completing treatment, B-hCG increased to 913 mIU/mL. VIP, etoposide, ifosfamide, and cisplatin. B-hCG, human chorionic gonadotropin

**Figure 9:**
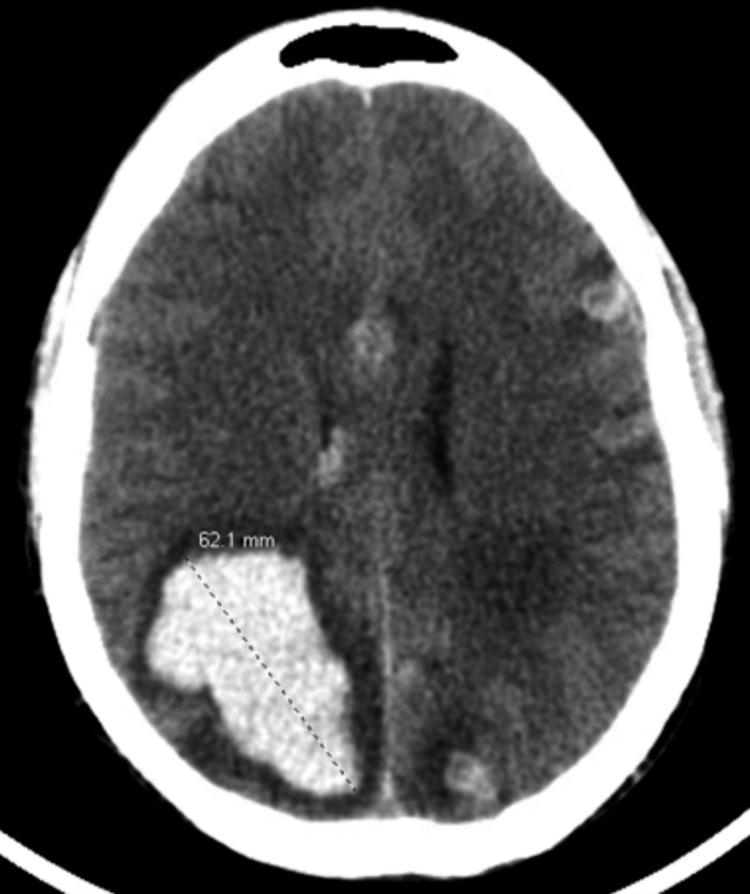
CT brain without contrast with new right intraventricular hemorrhage and multiple hemorrhagic metastases in bilateral cerebral hemispheres. Largest hemorrhagic lesion measuring about 6.2 cm in the right parietal lobe, increased size from prior imaging. Lesions demonstrating perifocal/vasogenic edema. CT, computerized tomography

A biopsy of a polypoid gastric lesion showed metastatic germ cell neoplasm. Notable labs on a presentation for normocytic anemia (Hgb 8.7 g/dL), severely elevated β-hCG of 185,667 mIU/mL, high lactate dehydrogenase of 598 unit/L, while alpha-fetoprotein (AFP) and thyroid studies were normal. Given the extent of the patient's metastatic testicular cancer, treatment was started immediately. However, with no residual disease on testicular ultrasound, the patient declined orchiectomy. The patient completed two cycles of bleomycin, etoposide, and cisplatin (BEP), then transitioned due to inability to tolerate bleomycin and completed four cycles of etoposide, ifosfamide, and cisplatin (VIP). After chemotherapy B-hCG levels decreased to 198 mIU/mL (Figure 7, 8). The patient was hospitalized two weeks after chemotherapy completion when several seizures, headaches, and non-bloody emesis occurred at home. Repeat brain imaging showed disease progression with multiple metastatic lesions increased in size with vasogenic edema with an active hemorrhagic component (Figure 9). β-hCG had increased to 913 mIU/mL during this hospitalization (Figure 8).

Electroencephalogram demonstrated multifocal spikes consistent with multiple intracranial lesions. The patient was to undergo whole brain radiation therapy, but ultimately patient expired from hemorrhagic shock due to the bleeding brain metastases before initiation of radiation therapy.

## Discussion

Testicular cancers arise from germ cells and are divided into seminomatous germ cell tumors (SGCT) and non-seminomatous germ cell tumors (NSGCT). NSGCTs are further classified into a choriocarcinoma, embryonal carcinoma, teratoma, and yolk sac tumors. Both embryonal carcinoma and choriocarcinoma in their pure form are quite rare. Our patient's tumor showed a mixed tumor on histological examination comprising embryonal carcinoma and choriocarcinoma. Both histologic tumor types are known to exhibit testicular tumor regression, known as a "burned-out" tumor. The primary site of choriocarcinoma is commonly in the testis or anterior mediastinum. Extragonadal origin sites must be considered if the testicular exam and ultrasound are negative. Patients with widespread metastatic disease may have no residual primary tumor or detectable mass in the testicle with a "burnt-out" appearance. At this stage, testis imaging can show scar tissue and calcifications. With no sign of a primary testicular source, it behooves the clinician to search for extragonadal sources in the brain, retroperitoneum, lungs, and liver.

Most testicular cancers are curable with appropriate early detection and therapeutic options. However, testicular choriocarcinoma, given its aggressive and refractory nature, has a significantly lower rate of cure [1]. Choriocarcinoma is a subtype of testicular cancer that can present with clinical features such as gynecomastia with tenderness and hemoptysis [1]. Complications of choriocarcinoma have been documented in previous literature, with extensive hematogenous spread resulting in intracranial bleeding, alveolar hemorrhage, respiratory failure, gastrointestinal bleeding, tumor lysis syndrome, and hyperthyroidism [1].

Choriocarcinomas produce high levels of B-hCG because the cells are differentiated into a trophoblastic phenotype [1]. Normal functioning testes and prostate gland produce low levels of B-hCG in healthy men [1]. B-hCG secretion and tumor marker elevation are more associated with choriocarcinoma [1]. High levels of B-hCG in males with or without testicular mass are highly suspicious of choriocarcinoma. Serum B-hCG level is a tumor marker to reflect underlying disease activity. Higher levels of B-hCG represent a higher likelihood of multi-organ involvement with widespread metastases [1]. Unlike other testicular malignancies, choriocarcinoma should be treated immediately, even before a tissue diagnosis [1].

The immediacy and targeted treatment of choriocarcinoma are essential to control the burden of the rapidly growing metastatic disease. A young male patient with a solid testicular mass should undergo early radical orchiectomy as a diagnostic and treatment tool for testicular cancer. However, radical orchiectomy with evidence of an already regressing "burnt-out" testis tumor is controversial, and a decision was made in our patient to treat the already known advancing metastatic testicular cancer. It has been shown that for patients with "burnt-out" tumors, despite no primary source seen on ultrasound, post-orchiectomy biopsy reveals evidence of malignancy [2]. This fact and Culine et al. suggest persistent testicular malignancy with extragonadal germ cell tumor can occur despite systemic chemotherapy [3]. Guidelines recommend that metastatic choriocarcinoma with the goal of tumor marker normalization should receive induction therapy with BEP. However, if the patient cannot tolerate bleomycin or is clinically unstable, the VIP regimen can be an alternative [4]. There have been suggestions for an individualized chemotherapy regimen tailored to the extent of the patient's disease. For example, a patient with multiple lung metastases at presentation should be administered etoposide and cisplatin without bleomycin as an induction regimen to avoid further respiratory compromise [5]. Using B-hCG as a disease activity marker, studies have shown that successful normalization of B-hCG below 10 mIU/mL indicates an excellent response to the treatment [6]. However, as in our case, the patient may display a refractory disease evident by higher plateau levels of B-hCG even after multiple rounds of chemotherapy. A male patient with high serum B-hCG with known lung, liver, and brain metastases with new symptomatology should raise suspicion for choriocarcinoma syndrome. New headaches, seizures, vision changes, hemoptysis, chest pain, or dyspnea could represent metastases in their respective organs [7].

Choriocarcinoma syndrome (CS) is an infrequent complication of testicular choriocarcinoma associated with poor outcomes. The cardinal feature of this syndrome involves massive hemorrhage from metastatic sites due to the rapidly evolving tumor burden that quickly overutilizes its local vascular supply. CS may be a sequela of either chemotherapy or presents as a spontaneous hemorrhage in advanced choriocarcinoma [8]. No diagnostic criteria exist to define choriocarcinoma syndrome. However, Logothetis et al. in 1984 described the syndrome as a striking elevation in B-hCG (over 50,000 mIU/mL) with an element of bleeding from metastatic sites of advanced testicular tumors [9]. Prior described cases include alveolar and gastrointestinal bleeding. Our patient with widespread pulmonary, stomach, hepatic, and brain metastases with B-hCG of over 100,000 mIU/mL developed worsening intracerebral hemorrhage of his brain metastases after completion of chemotherapy. The lack of alveolar hemorrhage or gastrointestinal symptoms, even after completion of chemotherapy, should not be overlooked in patients with aggressive choriocarcinoma. The clinical presentation of rapidly enlarging known brain metastases with hemorrhaging with symptoms of seizures, headaches, and vomiting is compatible with the diagnosis of choriocarcinoma syndrome. Reports of testicular choriocarcinoma of the brain resulting in intracerebral hemorrhage are limited. The prognosis for CS is poor due to inherent hemodynamic instability with actively hemorrhaging sites. Pathogenesis of CS is unclear, but it has been hypothesized that the cytotoxic chemotherapy regimen may be directly responsible for activating spontaneous bleeding [7]. Another possible trigger for CS is tumor cell lysis due to chemotherapy, which leads to tumor invasion of blood vessels [10]. Treatment for brain metastases in the setting of metastatic choriocarcinoma is sparse. Hemorrhage is the leading cause of death in patients with testicular choriocarcinoma [11]. The treatment approach for choriocarcinoma with brain metastases usually includes chemotherapy, radiotherapy, or in particular cases, surgery. The crux of managing choriocarcinoma would be first recognizing the constellation of symptoms and preventing further damage, such as managing respiratory failure and refractory gastrointestinal bleeding while aggressively treating choriocarcinoma.

Utility of ChatGPT in medical literature writing

Artificial intelligence (AI) is constantly evolving, especially in healthcare technology. An example of AI making its debut in scientific literature is known as, ChatGPT. This AI tool is a language generation model in the form of a chatbot that can interpret and respond to inquiries in a conversational format. It is used in various applications, including content creation by generating blogs from keywords, reducing workload by automating customer service responses or even passing the U.S. Medical Licensing Examination (USMLE) [12].

**Figure 10:**
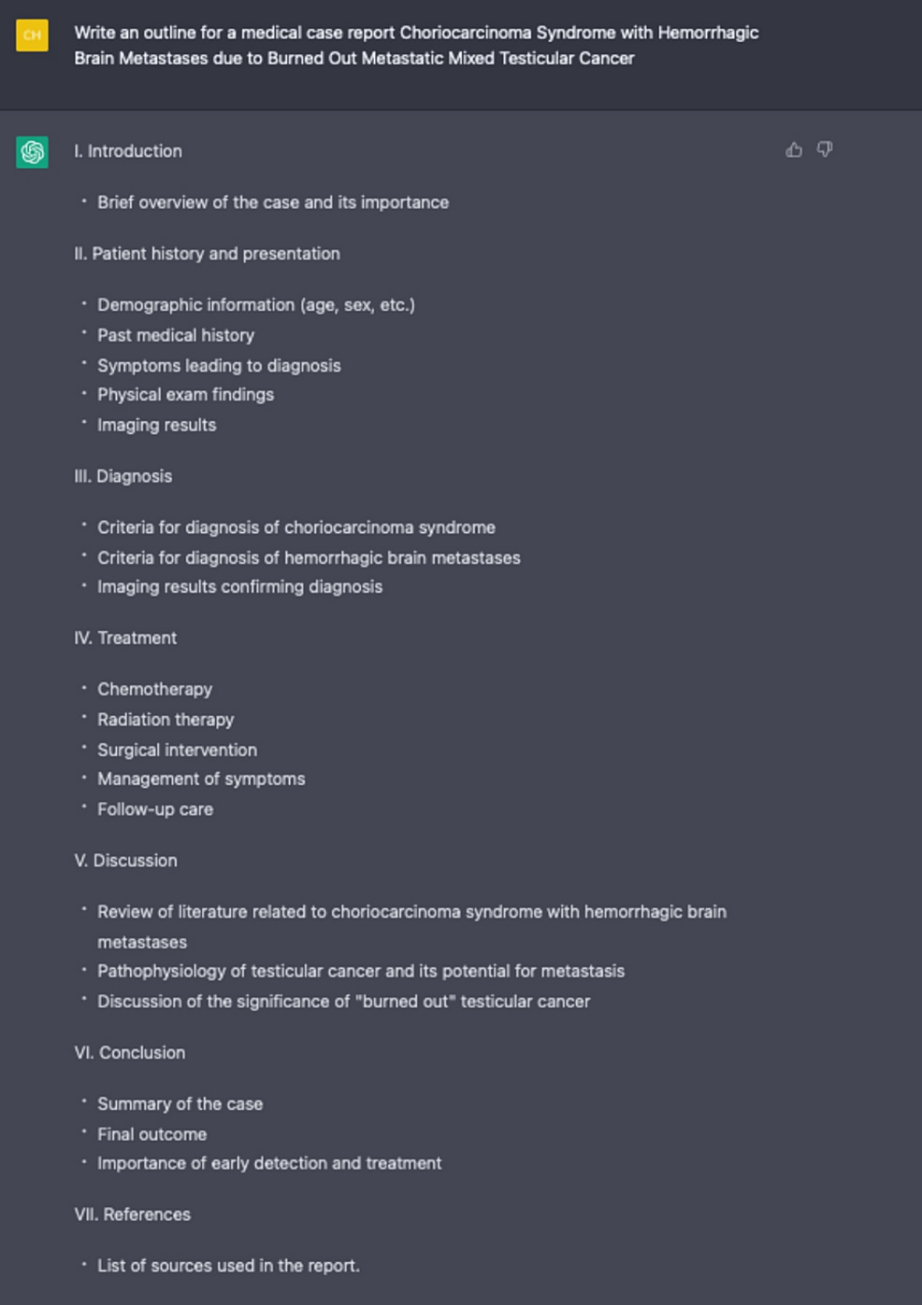
Using ChatGPT to outline this article with specific instructions.

While using ChatGPT, we were exhilarated to explore the potential uses of creating content for our clinical vignette. We first asked ChatGPT to generate an outline to serve as the foundation and to organize our approach to the report. The first step was easy, as in any natural conversation, to start with an inquiry or direction. ChatGPT generated a cohesive and logical response that would aid in organizing a case report (Figure 10). We filled in the details under each outlined section that ChatGPT generously provided.

**Figure 11:**
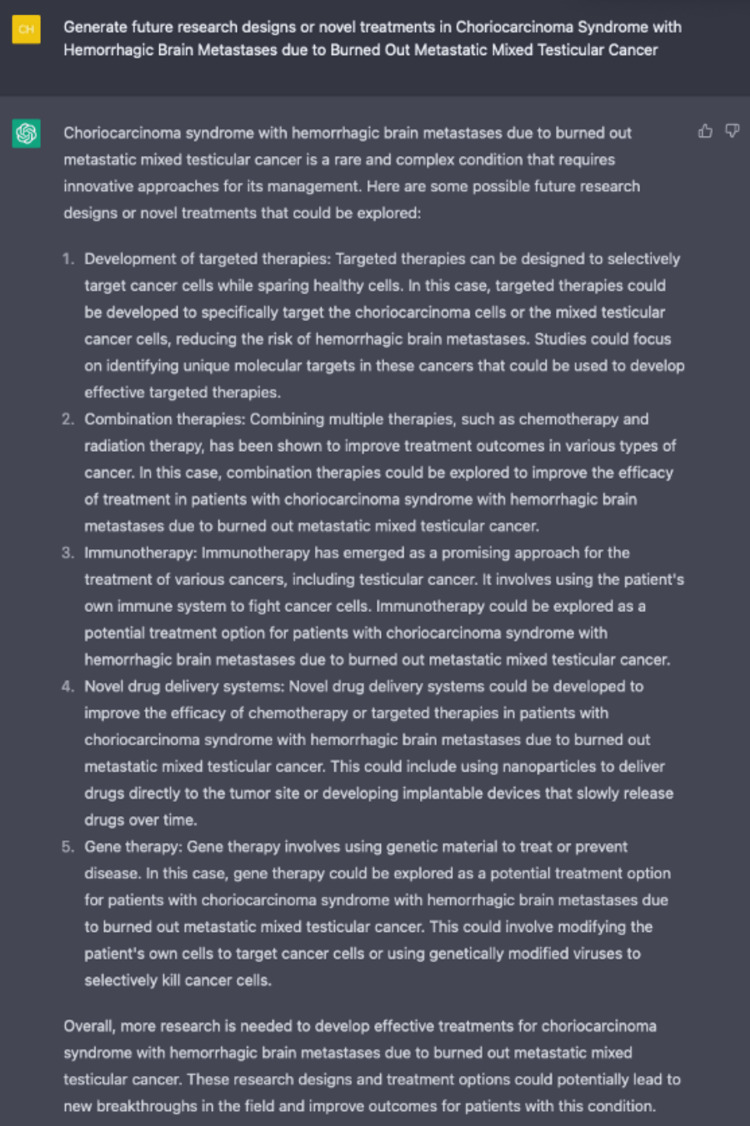
Generation of future areas of research by ChatGPT.

We also asked ChatGPT what other research designs or novel treatments future research should be conducted (Figure 11). To our surprise, ChatGPT generated a great list of future directions researchers can explore, such as targeted and immunotherapy for testicular cancer cells.

**Figure 12:**
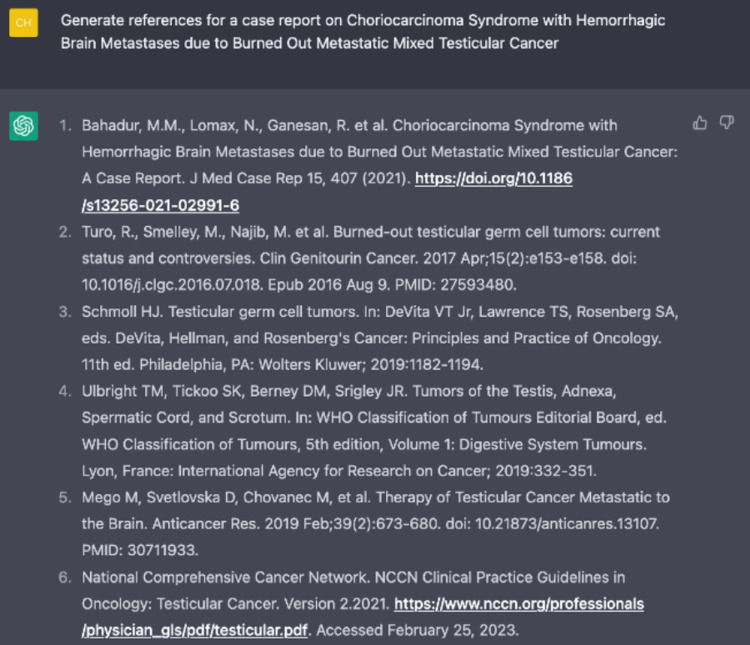
Generated list of references from ChatGPT with generic formatting instructions.

In addition, we asked ChatGPT to form a bibliography with references in a corrected format. However, it became obvious that ChatGPT's glaring weakness was the inaccuracies found within the list of automated references. The first few citations were linked to articles that did not exist. The PubMed IDs (PMIDs) and Digital Object Identifier (DOI) were randomly generated, and no articles were found in our search. The particular use case for ChatGPT would be for writers to input papers with correct PMIDs and DOIs manually and have ChatGPT format it in the requested style for each journal. ChatGPT, as a scientific formatting tool, is free compared to expensive citation tools such as EndNote. The rest of the included references were generic books or guidelines that were generated based on keywords rather than specific content that can be referenced in the report (Figure 12). At first glance, the ability of ChatGPT to generate a cohesive, concise, and professional response can lead to misinformation if verified and analyzed correctly. Instead, the particular strengths of ChatGPT should be more attuned to organizational and idea-generation purposes.

ChatGPT is a groundbreaking medical writing literature tool that can be used to enhance and support the work of researchers. ChatGPT, at its fingertips, has a powerful ability to parse insights from large pools of data from the internet and can generate research designs or areas of research opportunities in the future. ChatGPT design is intuitive, and with its ease of use, any writer can benefit from ChatGPT's computational abilities. Users may find it easier to use ChatGPT if a specific inquiry is made. A refined and discrete question or statement will yield better results using ChatGPT. With more fine-tuning from user input, ChatGPT can be an invaluable tool for writing complex medical literature. However, in its current state, ChatGPT should not be used to substitute a human clinical experience, nor should it be capable of fully understanding the nuances and intricacies of clinical cases or research studies.

## Conclusions

Our case illustrates a rare choriocarcinoma syndrome with intracerebral hemorrhaging from brain metastases in a patient with burned-out mixed testicular cancer (embryonal and choriocarcinoma). Clinicians who first encounter a young male patient with systemic involvement should be aware of choriocarcinoma syndrome's rapid and fatal complications. Despite a negative testicular physical exam or testicular ultrasound, emergent treatment should not be held for orchiectomy, diagnostic work-up, or biopsies. CS can be a diagnostic challenge without a primary testicular tumor mass or testicular symptoms. However, early referral to a more experienced center to treat advanced germ-cell tumors should be considered. ChatGPT has broad usages in assisting with medical literature writing for specific scenarios. Generating an outline, proofreading sections, affordability of citation management, and producing novel ideas and treatments for future research are all ways ChatGPT can streamline the writing process for authors. However, the danger of solely relying on the chatbot due to falsifying texts legibly will challenge how journals in the future will accept the usage of ChatGPT. Legitimate concerns have been raised about the accuracy, integrity, and acceptance of ChatGPT as a medical literature tool. We advocate that using the generational language model can assist writers but not supplant them.
